# Acute Toxicity of Three Synthetic Cannabinoids: First In Vivo Preclinical Study

**DOI:** 10.3390/molecules31132365

**Published:** 2026-07-05

**Authors:** Silviu-Iulian Filipiuc, Carmen Solcan, Bogdan-Ionel Tamba, Leontina-Elena Filipiuc, Veronica Bild, Daniela-Carmen Ababei, Gabriela-Dumitrița Stanciu, Maria-Raluca Gogu, Cristina-Mariana Uritu, Cezar Ilie Foia, Walther Bild

**Affiliations:** 1 Advanced Research and Development Center for Experimental Medicine “Prof. Ostin C. Mungiu”—CEMEX, Grigore T. Popa University of Medicine and Pharmacy, 700115 Iasi, Romania; silviu.filipiuc@umfiasi.ro (S.-I.F.); leontina.filipiuc@umfiasi.ro (L.-E.F.); veronica.bild@gmail.com (V.B.); daniela-carmen-p-ababei@umfiasi.ro (D.-C.A.); gabriela-dumitrita.stanciu@umfiasi.ro (G.-D.S.); raluca.gogu@umfiasi.ro (M.-R.G.); cristina-mariana.uritu@umfiasi.ro (C.-M.U.); 2 Faculty of Veterinary Medicine, “Ion Ionescu de La Brad” University of Life Sciences, 700490 Iasi, Romania; carmen.solcan@iuls.ro; 3 Department of Pharmacology, Clinical Pharmacology and Algesiology, Grigore T. Popa University of Medicine and Pharmacy, 700115 Iasi, Romania; foia.cezar-ilie@d.umfiasi.ro; 4 Pharmacodynamics and Clinical Pharmacy Department, Grigore T. Popa University of Medicine and Pharmacy, 700115 Iasi, Romania; 5 “Olga Necrasov” Center of Anthropological and Biomedical Research, Romanian Academy, Iasi Branch, 700506 Iasi, Romania; walther.bild@umfiasi.ro; 6 Department of Physiology, Grigore T. Popa University of Medicine and Pharmacy, 700115 Iasi, Romania

**Keywords:** synthetic cannabinoids, JWH-007, AM-694, MAB-CHMINACA, acute in vivo toxicity, synthetic cannabinoids with abuse potential, new psychoactive substances

## Abstract

Background and Objectives: Synthetic cannabinoids (SCs) are new psychoactive substances associated with acute intoxications. Experimental data obtained under controlled and comparable conditions remain limited for this category of compounds. This descriptive, hypothesis-generating screening study aimed to characterize the acute toxicity profile of three SCs, JWH-007, AM-694, and MAB-CHMINACA. Materials and Methods: Acute toxicity was evaluated in female Swiss Albino mice, in accordance with the OECD 423 guideline, following oral and intraperitoneal administration. Animals were monitored for 14 days for behavioral and clinical signs of toxicity. At the end, histopathological examination was performed to describe organ-level changes. Serum concentrations of the tested compounds were quantified by LC-ESI-MS/MS 24 h after intraperitoneal administration. Results: The three compounds were associated with distinct behavioral, clinical, and histopathological observations. JWH-007 was associated with transient behavioral depression and histopathological changes in peripheral organs. AM-694 was associated with histopathological changes in systemic organs and limited behavioral manifestations. MAB-CHMINACA was associated with acute behavioral toxicity and central nervous system lesions, including neuronal vacuolization, necrosis, oedema, and inflammatory changes. Conclusions: These preliminary findings describe compound-specific in vivo toxicity patterns and may inform the design of future confirmatory studies on SCs’ toxicity. The observed behavioral and histopathological changes should be interpreted as hypothesis-generating and require statistical validation before conclusions can be drawn regarding comparative toxicity, structural class effects, or predictive value for risk stratification.

## 1. Introduction

The human endocannabinoid system (ECS) represents one of the most widespread neuromodulatory signaling networks in the body, consisting of cannabinoid receptors CB1 and CB2, endogenous ligands, mainly anandamide (AEA) and 2-arachidonoylglycerol (2-AG), and their metabolic enzymes [1,2]. CB1 receptors, among the most abundant G-protein-coupled receptors in the central nervous system (CNS), are preferentially distributed in motor and limbic regions and in areas involved in pain modulation, while CB2 receptors are predominantly found in peripheral immune tissues, with maximum density in the spleen [3,4]. Through these receptors, the ECS regulates key physiological processes such as appetite, pain perception, immune function, inflammation, and seizure activity, which has led to significant interest in the therapeutic potential of cannabinoids. The approval of nabiximol, a Δ9-tetrahydrocannabinol (THC) and cannabidiol (CBD) mixture, for spasticity and neuropathic pain in multiple sclerosis, as well as a purified botanical cannabidiol for refractory forms of pediatric epilepsy, has strengthened the clinical relevance of this pharmacological class and brought ECS to the forefront of modern neuropsychopharmacology [5,6,7]. However, the psychoactive properties of THC, mediated through CB1 agonism in limbic and prefrontal regions, are associated with a recognized risk of abuse, dependence, and psychiatric vulnerability, and the progressive legalization of cannabis in many jurisdictions has amplified public health concerns related to recreational use of this class of compounds [8,9,10,11]. The overlap of the aforementioned therapeutic interest and the public availability of CB1-agonist structures have given rise to one of the most challenging issues in modern clinical toxicology: the systematic exploitation of cannabinoid receptor chemistry to produce new psychoactive substances (NPSs) with significantly higher potency than THC [8,12,13]. With 254 synthetic cannabinoids (SCs) monitored in the European Union’s early warning system by the end of 2023 and additional 20 new compounds notified in 2024, this class continues to represent the largest and most used and most dynamic group of illicit NPSs across Europe and worldwide [12,13]. SCs primarily function as full agonists of cannabinoid receptors, unlike THC, which serves as a partial agonist [14]. Their reported affinities are up to 100-fold greater than those of THC, a qualitative distinction that directly correlates with the clinical severity of intoxications [15]. The clinical manifestation of acute intoxication significantly exceeds the symptomatology associated with cannabis-based products, involving tachycardia with arrhythmic potential, hypertension, tonic-clonic seizures, psychotic agitation, hyperthermia, rhabdomyolysis, and acute renal failure [10,11]. Documented fatalities directly linked to the consumption of these compounds have been reported in Europe and North America in association with various structural classes [16].

Structurally, SCs have evolved in successive stages, each marked by increased potency at the CB1 receptor and increasingly poor preclinical toxicological documentation at the time of market entry [9,12]. The first class, represented by the JWH series of compounds, synthesized by John W. Huffman’s group, included naphthoylindoles with nanomolar affinities for CB1 and CB2 receptors [17]. Of these, JWH-007 (1-pentyl-2-methyl-3-(1-naphthoyl) indole), one of the most active compounds of the initial class of N-alkyl naphthoylindoles reported by Huffman’s group in 1994, exhibits CB1 receptor affinity of 9.50 nM, and was subsequently identified as an ingredient in “Spice” and “K1” products, and also in so called “herbal mixtures” on the European illicit drug market [17,18,19]. The AM series of compounds developed by Alexandros Makriyannis’ group introduced fluorinated substitutions on the N-alkyl chain, conferring increased lipophilicity, a distinct toxicokinetic profile and prolonged persistence of CB1 activation [8,19]. AM-694 (1-(5-fluoropentyl)-3-(2-iodobenzoyl) indole) is a selective CB1 agonist with high affinity (Ki = 0.08 nM) that has been reported in human consumption cases and detected analytically in biological samples, confirming its bioavailability and potential for intoxication by oral ingestion or smoking [8,20]. Similar to JWH-007, AM-694 has been identified as an adulterant in illicit herbal and drug-like products obtained via the Internet, together with other cannabimimetic compounds such as JWH-210, JWH-122 and JWH-019 [21]. MAB-CHMINACA (N-(1-amino-3,3-dimethyl-1-oxobutan-2-yl)-1-(cyclohexylmethyl)-1H-indazole-3-carboxamide), also called MAB-CHMINACA in alternative nomenclature, is a compound with high CB1 affinity (0.289 nM), which belongs to the class of indazole carboxamide compounds. This SC is responsible for multiple series of severe poisonings and documented deaths, including a series of 11 patients admitted to the emergency department with severe agitation, seizures and one death from anoxic brain injury and rhabdomyolysis, as well as three toxicologically confirmed postmortem deaths [22,23,24]. This SC has been reported in illicit herbal material products (“legal high”), including mixtures containing other synthetic cannabinoids such as 5F-AMB-PICA, EMB-FUBINACA and 5F-APP-PINACA [13].

This study was designed as a descriptive, hypothesis-generating screening investigation to obtain preliminary information on the acute toxicity of JWH-007, AM-694 and MAB-CHMINACA, three synthetic cannabinoids belonging to distinct structural classes and displaying different potencies as CB1 receptor agonists. Acute effects were explored after oral and intraperitoneal administration using the approach described in OECD Guideline 423, adapted in accordance with the 3Rs principles [25,26]. By its nature, this method classifies the tested substances into acute toxicity classes defined by fixed LD50 cut-off values, allowing a ranking of the three compounds with a minimum number of animals per dose [25]. The oral route reflects the most common scenario of recreational and accidental exposure, while the intraperitoneal route, associated with the determination of serum concentrations 24 h post-administration, constitutes a model of rapid systemic absorption, relevant to exposure by inhalation or injection, scenarios associated with the most severe onset of intoxication and the shortest therapeutic interval [8]. The main goal of this study was therefore to generate initial preclinical screening data for the acute toxicity classification of these three compounds, together with indicative toxicokinetic reference values obtained after intraperitoneal administration. Given the descriptive design and limited sample size inherent to this screening approach, all findings should be interpreted as preliminary. The observed toxicity patterns and comparative ranking are intended to support hypothesis generation only and require confirmation through statistically powered studies and more comprehensive toxicokinetic analyses. These preliminary data may nevertheless provide a useful starting point for interpreting clinical cases of acute intoxication and for designing future studies on recent-generation synthetic cannabinoids.

## 2. Results

### 2.1. Acute Toxicity Assessment Results

#### 2.1.1. Globally Harmonized System (GHS) Classification of the Three Synthetic Cannabinoids

**Figure 1:**
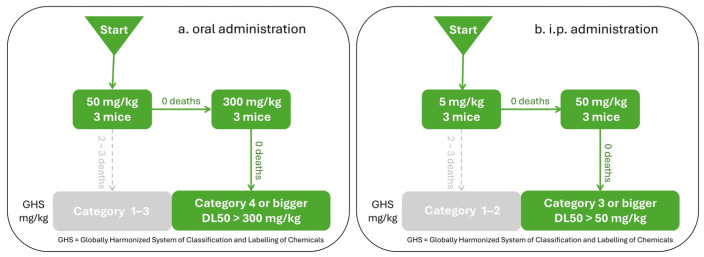
JWH-007, AM-694, MAB-CHMINACA LD50 estimation according to OECD Guideline 423 (Annex 2a for i.p. administration and Annex 2b for oral administration)—(**a**). oral administration and (**b**). i.p. administration.

Regarding the classification of the three SCs under the GHS, according to the OECD 423 guideline, they can be categorized as Class 4 or higher, with an LD50 > 300 mg/kg for oral administration (Figure 1b), and Class 3 or higher, with an LD50 > 50 mg/kg for i.p. administration (Figure 1a).

#### 2.1.2. Behavioral Assessment of the Three Synthetic Cannabinoids

##### Control Groups

The two control groups, with oral and i.p. administration, showed no evidence of toxicity within the first 24 h of administration. Furthermore, during the next 13-day secondary monitoring period, no significant changes in the animal’s overall health state were observed.

##### JWH-007

All behavioral manifestations observed following oral administration of JWH-007 are summarized in Table 1. Sedation was the most consistent behavioral effect after JWH-007 administration; however, some animals transiently exhibited motor incoordination, loss of balance, excitation, tremor, and occasional jumps during or between periods of sedation. At the 300 mg/kg dose, additional signs such as Straub tail, piloerection, and abnormal respiration were also observed.

Sedation was the predominant behavioral manifestation following i.p. JWH-007 administration, with transient motor incoordination and loss of balance occurring in some animals during or between sedative periods. The higher dose (50 mg/kg) induced a broader spectrum of neurological manifestations, including seizures, tremor, Straub tail, excitation, jumps, and head twitches. All behavioral manifestations observed following i.p. administration of JWH-007 are summarized in Table 2.

During the subsequent 13-day monitoring period, oral and i.p. administration of JWH-007 had no additional observable toxic effects on the animals’ health status.

##### AM-694

Within the first 24 h following oral or i.p. administration of AM-694, no behavioral signs of toxicity were observed or recorded in either of the groups. Additionally, during the next 13-day monitoring period, oral administration of AM-694 at doses up to 300 mg/kg and i.p. administration of AM-694 at doses up to 50 mg/kg had no observable toxic effects on the animals’ overall health status.

##### MAB-CHMINACA

All behavioral manifestations observed, following one dose of orally administrated MAB-CHMINACA at 50 mg/kg and 300 mg/kg, are summarized in Table 3. MAB-CHMINACA induced a more severe behavioral profile than JWH-007, characterized mainly by seizures, tremor, Straub tail, excitation with jumps, and sedation. Sedation occurred in alternation with intense neurological and motor manifestations and may therefore reflect, at least in part, a state of exhaustion following severe convulsive episodes and repeated motor disturbances rather than a purely depressant effect. At 50 mg/kg, MAB-CHMINACA produced a broader spectrum of adverse effects, including motor incoordination, loss of balance, writhes, loss of reactivity to touch, loss of grasping, catalepsy, loss of traction, and abnormal respiration. At 300 mg/kg, the observed manifestations were mainly seizures, tremor, Straub tail, sedation, and excitation with jumps.

During the next 13-day monitoring period, most monitored parameters remained within normal limits in both oral dose groups. In the 50 mg/kg group, reduced food intake was observed in 2 of 3 animals on day 1, while abnormal behavior, characterized by extreme docility and reduced attention paid to the surroundings, was recorded in 2 of 3 animals on days 4 and 7. In the 300 mg/kg group, reduced food intake was observed in 2 of 3 animals on day 2, and the same abnormal behavior was noted in 1 of 3 animals on days 2 and 3. No other relevant alterations were observed among the monitored parameters.

All behavioral manifestations observed following a single dose i.p. administration of MAB-CHMINACA at 5 mg/kg and 50 mg/kg are summarized in Table 4. Compared with oral administration, i.p. exposure induced a more intense convulsive profile, with marked neurological and motor manifestations and visible periods of exhaustion. At 5 mg/kg, MAB-CHMINACA produced seizures, tremor, Straub tail, sedation, excitation with jumps, motor incoordination, head twitches, loss of reactivity to touch, and catalepsy. Sedation was observed in alternation with convulsive episodes and motor disturbances, suggesting that it may reflect, at least in part, exhaustion following repeated neurological manifestations rather than a purely depressant effect. At 50 mg/kg, the behavioral profile was dominated by intense seizures, which alternated with tremor and Straub tail and persisted for up to 4 h. Due to the high intensity and persistence of these manifestations, other behavioral effects could not be reliably observed at this dose.

During the next 13-day monitoring period, one of the three animals in the 5 mg/kg i.p. group gained more than 10% body weight. In the 50 mg/kg i.p. group, two animals exhibited reduced food intake during the first two days post-administration.

### 2.2. Quantitation of Compounds by LC-ESI-MS/MS Results

**Figure 2:**
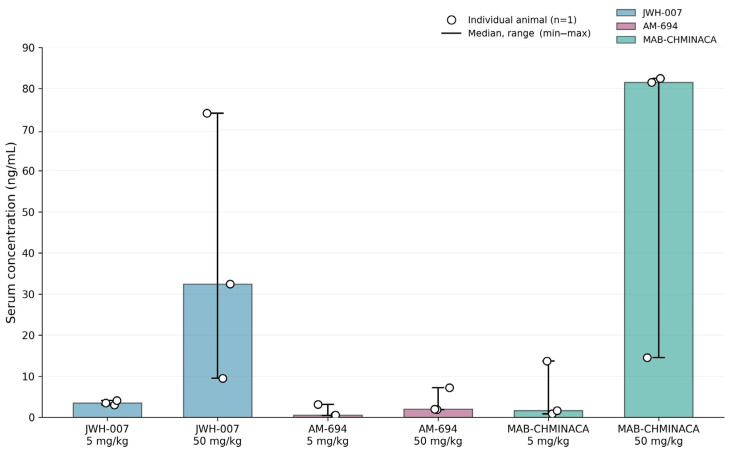
Data are shown as individual data points with median and minimum–maximum range (n = 3 per group).

For the 5 mg/kg dose, serum concentration levels of JWH-007 determined at 24 h after i.p. administration of each of the 3 SCs, using a LC-ESI-MS/MS method, ranged from 0.39 ng/mL to 4.11 ng/mL. For the 50 mg/kg dose, the serum levels ranged from 7.75 ng/mL to 67.62 ng/mL at 24 h after i.p. administration (Figure 2). For the 5 mg/kg dose, serum concentration levels of AM-694 measured using the same method at 24 h after i.p. administration ranged from 0.41 ng/mL to 3.16 ng/mL. For the 50 mg/kg dose, the serum levels ranged from 1.78 ng/mL to 7.24 ng/mL (Figure 2). For the 5 mg/kg dose, serum concentration levels of MAB-CHMINACA measured at 24 h after i.p. administration ranged from 0.83 ng/mL to 13.72 ng/mL. For the 50 mg/kg dose, the serum levels ranged from 47.26 ng/mL to 103.13 ng/mL (Figure 2). All results are presented as the mean ± the standard error of the mean.

### 2.3. Gross Necropsy and Histopathology Results

**Figure 3:**
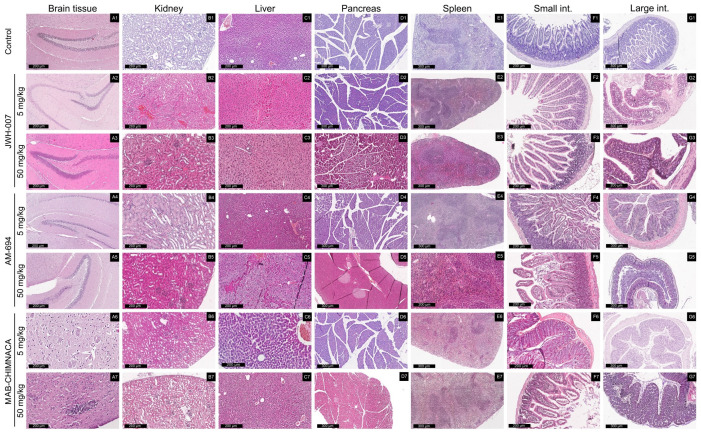
Histological examination of the brain, spleen, kidney, liver, pancreas, small and large intestine in mice exposed to 5 mg/kg and 50 mg/kg of SCs through i.p. administration. Histological analysis was performed using H&E staining. (**A1**–**G7**) subfigures details are in Table 5.

**Figure 4:**
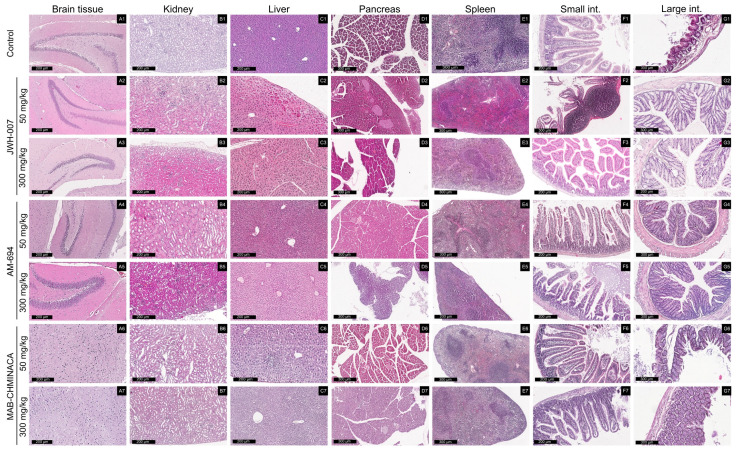
Histological examination of the brain, spleen, kidney, liver, pancreas, small and large intestine in mice exposed to 50 mg/kg and 300 mg/kg of SCs through oral administration. Histological analysis was performed using H&E staining. (**A1**–**G7**) subfigures details are in Table 6.

Histopathological changes observed after acute exposure to the three investigated SCs are summarized in Table 5 and Figure 3 for i.p. administration, and in Table 6 and Figure 4 for oral administration.

Overall, the lesions showed a compound-dependent pattern, with variable organ distribution and severity. Renal alterations were among the most frequently observed findings and included vascular congestion, tubular degeneration, acidophilic changes of the proximal tubules, and acute tubular necrosis.

Hepatic changes consisted of degenerative lesions, focal necrosis, pyknotic nuclei, and portal hypertension.

Pancreatic alterations were characterized by acinar changes with lipid inclusions, hypertrophy of the Langerhans islets, and vacuolated serous cells, while splenic lesions included vascular congestion, megakaryocyte proliferation, and stromal hyperplasia.

Intestinal alterations, represented by oedema and lymphoid infiltration, were observed in both the small intestine and, to a lesser extent, the large intestine (hypotrophy).

Notably, brain/CNS lesions, including small degenerative areas, cerebral necrosis, and vacuolar neuronal changes, were detected exclusively in MAB-CHMINACA-exposed groups, suggesting a distinct neurotoxic profile for this compound. No histopathological changes were observed in control animals.

**Figure 5:**
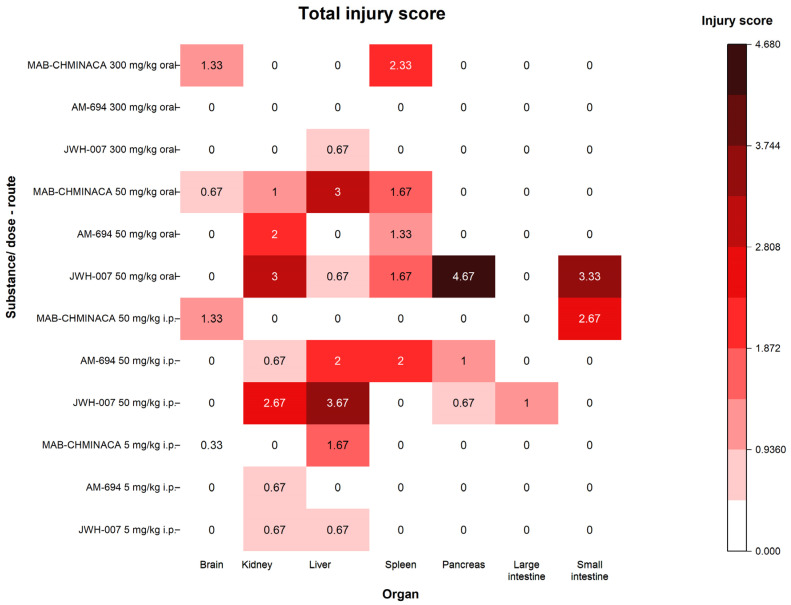
Heatmap of mean organ-specific injury scores (n = 3/group) for JWH-007, AM-694, and MAB-CHMINACA across tested doses and routes of administration. Scores reflect a five-point semi-quantitative histopathological scale (0–4), per INHAND recommendations.

The distribution of organ-specific injury scores across all dose/route/substance combinations is summarized in the heatmap shown in Figure 5, where each cell represents the mean injury score for a given organ-treatment group, with color intensity scaling from white (score = 0, no detectable lesions) to dark red (highest mean score), allowing direct visual identification of the organs most affected by each synthetic cannabinoid at each dose level.

For organs harvested from animals in which no lesions were detected (cerebellum, muscle, ovary, stomach, lung) in any of the 42 animals included in the study, separate composite figures were prepared and are available as part of the Supplementary Material (Supplementary Material Figures S1 and S2). Cardiac histopathology was not performed because terminal intracardiac blood collection precluded preservation of suitable heart tissue samples.

## 3. Discussion

The present study reports the first preliminary, descriptive preclinical screening data for JWH-007, AM-694 and MAB-CHMINACA, three synthetic cannabinoids belonging to different structural classes and with differing potencies as CB1 agonists. The scientific rationale for this study is based on several current gaps in the available evidence. First, comprehensive acute toxicity data are lacking for JWH-007, AM-694 and MAB-CHMINACA, and, to our knowledge, there are no formal estimates of toxicity classes or lethal-dose ranges obtained under controlled experimental conditions and comparable between distinct structural series. Second, available clinical and forensic reports mainly describe symptomatology and, in some cases, postmortem serum or tissue concentrations, but they do not correlate serum levels with the toxic picture under reproducible conditions. Therefore, the present work was intentionally framed as a preliminary, descriptive, hypothesis-generating screening study, designed to provide initial information on acute toxicity classification and indicative systemic exposure. Given the hypothesis-generating design and the limited number of animals per dose group, these findings should not be interpreted as statistically validated comparisons, but rather as exploratory observations requiring confirmation in adequately powered studies [24,27].

Recent studies in animal models have reported blood levels of AB-CHMINACA of 3.05–54.43 ng/mL one hour after repeated i.p. administration, providing a preliminary reference from a compound in the indazole-carboxamides class, but without correlation with the acute mortality profile or with data for structurally related compounds from different generations [28]. Third, the absence of comparative data between distinct structural series, naphthoylindoles (JWH), fluorinated benzoylindoles (AM) and indazole-carboxamides (CHMINACA) makes it impossible to estimate the relative severity of intoxication depending on the toxicologically identified compound. The results presented in this manuscript were obtained by oral and i.p. administration in accordance with the OECD 423 guideline adapted to the 3Rs principles and are supported by indicative, preliminary systemic exposure data. These results may serve as an initial framework for the interpretation of acute intoxication cases and as a foundation for comprehensive toxicokinetic studies of recent-generation SCs.

JWH-007 is a first-generation representative compound of naphthoylindoles, synthesized by Huffman’s group and subsequently identified in European “Spice” products. The compound exhibited a behavioral profile of predominantly depressant rather than excitatory effects in this descriptive screening model, consistent with the partial agonist pharmacology characteristic of this structural class at the CB1 receptor (Ki = 9.50 nM). Acute oral and i.p. administration yielded sedation, Straub tail reflex, and motor incoordination at both 50 mg/kg doses, with aggressiveness emerging only at the highest oral dose (300 mg/kg), a pattern qualitatively similar to that observed for the structurally analogous JWH-182, another naphthoylindole, when evaluated under the same protocol [29]. The behavioral phenotype partially reflects the documented tetrad effect (hypothermia, analgesia, hypolocomotion, catalepsy) characteristic of naphthoylindole agonists, which, like Δ9-THC, are partial rather than full CB1 agonists [30]. However, the 13-day post-dosing observation revealed full behavioral recovery with no sustained abnormalities. Histopathologically, JWH-007 demonstrated predilection for organs involved in drug metabolism and excretion, particularly the kidney, liver, and pancreas. The renal degenerations (acute tubular necrosis, vascular congestion) are consistent with reports in the literature of naphthoylindole-mediated nephrotoxicity, attributed in part to CB1-mediated disruption of renal hemodynamics and glomerular filtration [31]. While clinical reports of JWH-007 intoxication are scarce, case studies of first-generation naphthoylindoles such as JWH-018 and JWH-073 document behavioral symptoms ranging from sedation and lethargy to agitation, hallucinations, and in severe cases, seizures [32]. However, such severe neurological complications appear to be dose-, individual vulnerability-, and product-composition-dependent, and were not universally observed in preclinical studies. The present result for JWH-007 thus reflects a moderate systemic hazard profile, wherein toxicity is manifest principally through organ dysfunction rather than CNS damage, and behavioral recovery is achievable at sub-lethal doses. However, this interpretation remains preliminary and requires statistical and mechanistic validation. The present result for JWH-007 thus suggests, within the limitations of this screening design, a moderate systemic hazard profile, wherein toxicity was manifest principally through organ dysfunction rather than irreversible CNS damage. This conclusion should be regarded as preliminary, because the study design does not allow formal statistical confirmation of organ-specific susceptibility or recovery patterns.

AM-694 is a member of the AM series of SCs developed by Makriyannis’ group and characterized by a fluorinated N-alkyl chain and selective CB1 agonism with the highest affinity among the three tested compounds (Ki = 0.08 nM). This agent presented an apparently divergent behavioral phenotype in the present screening study: no observable toxic effects were recorded during the acute 24 h observation period or throughout the 13-day monitoring phase, despite doses up to 50 mg/kg i.p. and 300 mg/kg oral. This absence of visible behavioral toxicity stands in notable contrast to the substantial behavioral effects observed with structurally related AM-series compounds. AM-2201, produced, at systemic doses of 0.1–1.0 mg/kg (subcutaneously in rats), dose-dependent and pronounced catalepsy, hypothermia, and immobility lasting 4–8 h postinjection [33]. The apparent discrepancy between limited observable behavioral manifestations and systemic organ involvement in AM-694 remains unexplained by the present dataset. The single 24 h serum measurement does not allow conclusions regarding pharmacokinetic profiles, blood–brain barrier penetration, or CNS exposure, which require dedicated toxicokinetic and brain distribution studies. This proposed explanation is speculative and requires dedicated toxicokinetic, receptor-binding, and tissue-distribution studies for validation. AM-2201 achieves peak plasma concentrations of 0.14–67.9 μg/L at 1–2 h post-administration [33]. In contrast, AM-694’s rapid tissue redistribution due to its high lipophilicity, conferred by the fluoropentyl substitution, which increases partition coefficient by ~2-fold relative to non-fluorinated congeners, may result in rapid sequestration into peripheral adipose and hepatic compartments. This hypothesis is supported by the clinical case of AM-694, the first documented in vivo human exposure, wherein the parent compound was detected exclusively in urine and not in serum, despite intoxication symptoms (trauma with alcohol co-ingestion), suggesting that either the brief 24 h sampling window missed the acute phase, or that hepatic first-pass metabolism and rapid tissue sequestration limits sustained CNS bioavailability [34]. Histopathologically, AM-694 induced pancreatic toxicity, characterized by acinar hyperplasia, hypertrophied Langerhans islets, and focal fatty changes, coupled with modest hepatic degeneration and renal vascular congestion, but conspicuously absent were CNS lesions of any severity. The pancreatic findings are particularly noteworthy and mechanistically intriguing: CB1 and CB2 receptors are expressed on islet β-cells and hepatic stellate cells, and CB1 activation has been shown to impair glucose homeostasis and promote lipid accumulation [35]. Thus, the islet hyperplasia may represent a compensatory response to cannabinoid-induced metabolic dysregulation rather than direct β-cell necrosis. The limited observable behavioral manifestations recorded for AM-694, despite evidence suggesting systemic organ involvement, remain descriptive observations that cannot be mechanistically interpreted on the basis of the present dataset. The present study did not assess receptor engagement, multi-time-point toxicokinetic, tissue distribution, or brain concentrations; therefore, no conclusions can be drawn regarding CNS exposure, blood–brain barrier permeability, or peripheral versus central CB1 involvement.

MAB-CHMINACA is classified within the indazole carboxamide structural class, being characterized by exceptionally high CB1 affinity (Ki = 0.289 nM) coupled with full agonist efficacy at CB1 receptors. The compound produced marked acute neurobehavioral signs in this screening model at all doses by both oral and i.p. routes, manifested with a fulminant behavioral syndrome dominated by convulsions, Straub tail reflex, loss of righting reflex, loss of reactivity to touch, loss of muscle tone, and catalepsy within 15–30 min after administration. These findings indicate a concerning toxicity pattern, but their relative severity compared with the other compounds remains preliminary and requires statistical validation. This complex behavioral phenotype may reflect, as a mechanistic hypothesis, the pharmacological distinction between partial and full agonists: while JWH-007, a partial agonist naphthoylindole, induced seizures only at higher doses (50 mg/kg i.p.) and with a lower intensity and frequency, MAB-CHMINACA, as a full CB1 agonist, achieved maximal signaling at lower doses and triggered rapid excitatory cascades. However, the present study was not designed to directly test this mechanism, and therefore this interpretation should be treated as exploratory.

Histopathologically, MAB-CHMINACA demonstrated CNS parenchymal alterations in the examined animals, including small degenerative areas with neuronal swelling at 5 mg/kg i.p, and cerebral necrosis with vacuolar neuronal changes and oedema at 50 mg/kg i.p. These observations suggest potential CNS vulnerability, but the frequency and severity of these lesions require confirmation in larger, statistically powered cohorts. Thus, within the limits of this descriptive screening study, MAB-CHMINACA showed a neuropathological pattern that appeared qualitatively different from those observed for JWH-007 and AM-694. This distinction should not be interpreted as a statistically established ranking of toxicity, but as a preliminary observation requiring validation.

The clinical correlate is evident from the 2014 MAB-CHMINACA outbreak reported by Katz et al., wherein 11 patients presented to a single emergency department with life-threatening toxicity: 9 required intubation, 3 experienced seizures, and 1 developed anoxic brain injury and rhabdomyolysis leading to death [23]. Blood concentrations in these patients ranged from 1.3 to 14.6 μg/L within 1–2 h of intake, a range directly predictive of the GHS Class 3 (i.p.) classification (LD50 > 50 mg/kg) derived in the present study; furthermore, postmortem femoral vein blood concentrations in a fatal MAB-CHMINACA case were 6.1 μg/L, with accumulation in liver (156 ng/g) and kidney (131 ng/g) due to the high lipophilicity of the indazole carboxamide scaffold [36]. The severe neurological manifestations, e.g., seizures, rhabdomyolysis (indicating myopathy secondary to prolonged muscle rigidity and excitation), and anoxic brain injury, observed clinically, are thus quantitatively concordant with the behavioral manifestations and neuronal necrosis documented preclinically.

Taken together, these findings suggest that, in this exploratory model, the toxicological profile of SCs may not be inferred solely from their formal GHS acute toxicity class or from receptor affinity considered in isolation. Although JWH-007, AM-694, and MAB-CHMINACA were assigned to broadly comparable GHS categories, their biological effects appeared to differ descriptively in terms of clinical expression, peripheral organ involvement, CNS injury, and reversibility. Because this study was not powered for statistical comparison, these apparent differences should be regarded as hypothesis-generating rather than definitive.

The pattern observed in the present study supports the hypothesis that naphthoylindole-type partial agonism was associated mainly with reversible neurofunctional depression and peripheral organ toxicity. Fluorinated indole exposure produced a dissociation between minimal observable behavioral signs and systemic organ damage, whereas indazole carboxamide full agonism was linked to the most severe phenotype, characterized by acute neurological toxicity and direct CNS parenchymal injury. These proposed relationships remain preliminary and require confirmation through statistically powered studies incorporating quantitative behavioral, biochemical, and toxicokinetic endpoints.

The dissociation observed between CB1 receptor affinity and in vivo toxicity further suggests, but does not prove, the need for a broader pharmacological and toxicokinetic interpretation of acute SC poisoning. AM-694, despite having the highest CB1 affinity among the tested compounds, did not induce observable behavioral toxicity under the present experimental conditions, suggesting that affinity alone may be insufficient to predict clinical severity. This conclusion is preliminary and should be validated using larger cohorts and direct measurements of CNS exposure, receptor engagement, and functional outcomes.

These observations have potential implications for clinical toxicology, forensic interpretation, and postmortem investigation. In emergency settings, the absence of marked behavioral or neurological manifestations should be interpreted cautiously and should not automatically be taken as evidence of absent systemic toxicity, as illustrated by the preliminary AM-694 profile, in which organ-level injury occurred despite minimal observable behavioral changes. Conversely, severe neurological manifestations such as seizures, loss of righting reflex, catalepsy, or altered responsiveness may raise suspicion for exposure to full-agonist indazole carboxamides, especially when supported by biochemical evidence of systemic injury. These translational implications remain exploratory and require validation against clinical and forensic datasets.

A notable limitation of this study is the small sample size inherent to the OECD Guideline 423 design, which prescribes a minimum of three animals per dose step in accordance with the 3Rs principles of animal research. While this approach is ethically and methodologically justified, it precludes formal statistical analysis, and consequently, the histopathological findings reported herein, although biologically plausible and internally consistent, cannot be subjected to tests of statistical significance. Therefore, all organ- and CNS-level toxicity patterns described in this manuscript should be interpreted as preliminary, descriptive, and hypothesis-generating. An additional limitation concerns the qualitative nature of the behavioral assessment. Behavioral monitoring was performed as a binary presence or absence screen adapted to the OECD 423 design, rather than as a standardized ordinal scoring system. Consequently, the present data do not allow semi-quantitative assessment of behavioral intensity, graded severity, or dose–response relationships. The behavioral findings should therefore be interpreted only as preliminary descriptive observations. Future studies should incorporate standardized ordinal scoring systems for key behavioral parameters, together with adequately powered statistical analyses.

The present study also highlights a limitation of applying conventional GHS-based acute toxicity classification to SCs without additional mechanistic descriptors. The GHS system is useful for estimating lethality-based hazard, but in this exploratory dataset it did not fully capture qualitative differences between reversible pharmacological effects and irreversible CNS parenchymal injury.

Finally, the concordance between the behavioral and histopathological findings observed in this experimental model and previously reported clinical manifestations suggests possible translational relevance of the mouse acute toxicity model for the preliminary evaluation of emerging SCs. However, this relevance remains provisional, and several limitations should be acknowledged. The present study employed oral and intraperitoneal administration in mice, whereas human exposure most commonly occurs through inhalation or ingestion, often in the context of unknown doses, product mixtures, and co-exposures. Future studies using exposure routes that more closely reproduce human consumption patterns, together with extended behavioral follow-up, advanced neuropathological techniques, and real-time functional assessment of CNS injury, would further clarify the mechanisms underlying SC toxicity and improve the predictive value of preclinical testing.

## 4. Materials and Methods

### 4.1. Reagents

SCs JWH-007 (CAS: 155471-10-6, Item No. 10266), AM-694 (CAS: 335161-03-0, Item No. 10567), and MAB-CHMINACA (CAS: 1863065-92-2, Item No. 16616) were purchased from Cayman Chemical (Ann Arbor, MI, USA). Sodium carboxymethyl cellulose (Na-CMC) was supplied by Alfa Aesar GmbH & Co., Ltd., (Kandel, Deutschland); Tween 80 by Sigma-Aldrich (USA); and 0.9% saline solution by B. Braun Pharmaceuticals S.A. (Timișoara, Romania). Isoflurane (ISOFLUTEK 1000 mg/g) used for animal anesthesia was obtained from Laboratorios KARIZOO, S.A. (Caldes de Montbui, Barcelona, Spain).

Analytical standards for JWH-007, AM-694, MAB-CHMINACA, and JWH-018-d9 were obtained from Cayman Chemical in crystalline form. Acetonitrile was purchased from VWR Chemicals (Rosny-sous-Bois, France) and was of HPLC, UHPLC, and LC-ESI-MS/MS grade. Methanol was sourced from LAB-SCAN Analytical Sciences (Gliwice, Poland) and was of HPLC grade. Formic acid 99.7% was obtained from Merck (Deutschland). Ultrapure water (0.055 μS/cm conductivity) was produced using a UV Atrium Mini Plus system (Sartorius Lab Instruments GmbH & Co., Ltd., Goettingen, Deutschland).

All reagents for tissue fixation, processing, embedding, and staining were purchased from Richard-Allan Scientific (Kalamazoo, MI, USA) and used according to the manufacturer’s specifications.

### 4.2. Acute Toxicity Assessment

A total of 42 healthy female Swiss Albino mice, aged 8–12 weeks, nulliparous, and non-pregnant, weighing between 20 and 40 g, were obtained from Cantacuzino Institute (Bucharest, Romania). Also, the animals were not genetically modified, had no previous experimental procedures, and had no known health or immune abnormalities at the time of inclusion. The mice were housed in the animal research facility of the Advanced Research and Development Center for Experimental Medicine “Prof. Ostin C. Mungiu”—CEMEX, Grigore T. Popa University of Medicine and Pharmacy Iasi, 700115 Iasi, Romania, under controlled environmental conditions (ambient temperature of 20 ± 4 °C, relative humidity of 50 ± 5%, and a 12 h artificial light/dark cycle, 07:00/19:00). They were kept in individually ventilated cages (IVCs), with three animals per cage, and had ad libitum access to food and water. The experimental study was authorized by the Research Ethics Committee (No. 638/23.09.2025) and by Romanian National Sanitary Veterinary and Food Safety Authority (No. 82/02.12.2025), and was conducted in accordance with European Directive 2010/63/EU and the ARRIVE guidelines [37,38].

The acute toxicity study was conducted via oral and i.p. administration according to OECD Guideline 423 (Annex 2a for i.p. administration and Annex 2b for oral administration), adapted in accordance with the 3Rs principles and the specific objectives of the study [25,26]. Groups of three animals were used per dose level; in the absence of mortality, dose escalation proceeded directly to the next level without repetition and was discontinued once sufficient evidence of toxicity was obtained. The aim was not to establish a precise LD50 interval and a category in the Globally Harmonized System of Classification and Labelling of Chemicals (GHS), but to identify the minimum dose associated with observable adverse effects based on behavioral and histopathological assessment. Accordingly, the animals were randomly allocated into 14 groups (n = 3 animals/group), using Random Team Generator (available at: https://www.randomlists.com, accessed on 20 May 2026). No a priori statistical power calculation was performed; the sample size of n = 3 per dose step was fixed by the step-down procedure of OECD Guideline 423. Animal/cage location and a non-toxic, wax-based color mark applied to the nape of each animal were used to avoid misidentification. Two groups served as controls: one vehicle control for i.p. administration and one vehicle control for oral administration. The remaining 12 groups were assigned to the three tested SCs, with four dose–route combinations per substance: 5 mg/kg i.p., 50 mg/kg i.p., 50 mg/kg orally, and 300 mg/kg orally (3 substances × 4 dose–route combinations). Female mice were used in accordance with OECD Test Guideline 423, which recommends a stepwise acute toxicity procedure using three animals of a single sex per dose step, normally females. This choice is based on the guideline’s rationale that testing in one sex, usually females, is considered sufficient for this acute toxicity classification approach, and that females are generally selected because, when sex-related differences are observed in conventional LD50 studies, females tend to be slightly more sensitive. No additional inclusion or exclusion criteria were applied during the experiment or data analysis, and all animals and administered experimental groups were included in the final analysis. The designated veterinarian responsible for monitoring the animals’ general health status was aware of group allocation, whereas the experimental personnel responsible for monitoring experiment-specific parameters and conducting the experimental procedures were blinded to group allocation. In addition, all experts involved in data interpretation, including the evaluation of experiment-derived data and histopathological analyses, were blinded to group allocation throughout the assessment process.

For oral administration, synthetic cannabinoids were prepared as a suspension using distilled water, Tween 80, and Na-CMC (98.9%; 1%; 0.1%). After formulation, they were administered in a volume of 0.20 mL/10 g body weight using size 8 gavage probes with rounded tips. For i.p. administration, the compounds were prepared as a solution using saline solution and Tween 80 (99%; 1%). After formulation, they were administered in a volume of 0.20 mL/10 g body weight using single-use syringes with a 0.01 mL graduation and appropriately sized needles.

The primary monitoring protocol and parameters followed were adapted from the Irwin Primary Observation Test for rodents [39]. After compound administration, each animal was continuously monitored during the first 15 min and then periodically at 30 min, 1 h, 2 h, 4 h, 8 h, and 24 h using a video tracking system and detailed monitoring sheets. The primary monitoring period focused on detecting parameters such as sedation, seizures, motor incoordination, loss of balance, tactile reactivity, and tremors. Over the subsequent 13 days, the animals were monitored daily for general health parameters, including lethality, morbidity or signs of illness, abnormal breathing or respiratory signs, abnormal appearance, abnormal behavior, analgesia, food intake abnormalities, water intake abnormalities, abnormal defecation, weight loss, weight gain, and body weight. Humane endpoint criteria were established a priori based on OECD guidance on the recognition and use of clinical signs as humane endpoints in safety evaluation studies and institutional animal welfare procedures [40]. Animals were to be humanely euthanized if they showed severe or persistent signs of distress, moribund state, marked reduction in responsiveness, severe respiratory distress, inability to access food or water, severe/prolonged convulsions, or marked body weight loss.

### 4.3. Quantitation of Compounds by LC-ESI-MS/MS

Serum concentrations of the three SCs were determined at 24 h after i.p. administration of each of the three SCs by liquid chromatography coupled with electrospray ionization tandem mass spectrometry (LC-ESI-MS/MS), using a method previously developed and validated by our team [29,41]. Briefly, blood was collected by retro-orbital sampling and serum was separated by centrifugation at 2000× *g* for 10 min, then stored at −24 °C until analysis. Protein precipitation was performed with ice-cold acetonitrile, followed by dilution with ultrapure water prior to injection, reducing the acetonitrile content below 50% to optimize chromatographic retention. Chromatographic separation was performed on a Kinetex C18 column (100 × 4.6 mm, 2.6 μm) using an acetonitrile/water gradient with 0.1% formic acid at a flow rate of 0.8 mL/min. The mass spectrometer was operated in positive ionization mode with a capillary voltage of 4000 V and a gas temperature of 325 °C. Quantification was performed in multiple reaction monitoring (MRM) mode using a deuterated internal standard for each compound. The method was validated for selectivity, linearity (0.5–1000 ng/mL), limit of quantification (LOQ 0.5 ng/mL), inter-run precision (CV% < 9%), and process efficiency, confirming its suitability for the quantification of SCs in serum matrix at toxicologically relevant concentrations. The results are expressed as the mean ± the standard error of the mean (SEM).

### 4.4. Gross Necropsy and Histopathology

A full necropsy was performed on all experimental animals following euthanasia by Isoflurane overdose. This included a thorough examination of the external surface of the body, all orifices, and the internal cavities and their contents. For each animal in the experiment, eleven organs were harvested and preserved in 10% neutral buffered formalin for histopathological examination, following the collection and fixation protocol previously described by our group [29,41]. Organ selection was guided by prior literature on the toxicity profile of SCs, prioritizing tissues most commonly reported as targets of toxicity, including pulmonary, hepatic, renal, and central nervous system components. After fixation, sections from these organs were processed into paraffin blocks, sectioned, and prepared for microscopic examination using hematoxylin–eosin staining. The prepared slides were scanned with a Leica Aperio digital microscope and then analyzed in detail to record structural changes and abnormalities. Histopathological lesions were graded semi-quantitatively according to INHAND guidelines and toxicologic pathology recommendations [42,43,44,45,46,47] using a five-point scale: 0 = no visible histopathological changes, 1 = minimal changes, 2 = mild changes, 3 = moderate changes, 4 = marked/severe changes [48]. Lesions were evaluated independently for each organ and animal by a histopathologist blinded to substance allocation. Organ-specific injury scores were subsequently calculated by summing the severity grades of all lesions identified within a given organ.

### 4.5. Statistical Analysis

Statistical analysis was not performed in this study, as the experimental design followed OECD Guideline 423, which prescribes a fixed group size of three animals per dose step in accordance with the 3Rs principles, precluding formal inferential statistics. Serum concentration data obtained by LC-ESI-MS/MS at 24 h post-administration are expressed as individual animal values with median and minimum–maximum range (n = 3 per group) and were calculated using OriginPro 2024 (OriginLab Corporation, Northampton, MA, USA). All other findings, including behavioral observations and histopathological results, are reported descriptively.

## 5. Conclusions

The present study provides preliminary, descriptive screening data on the acute toxicological profiles of JWH-007, AM-694, and MAB-CHMINACA. In accordance with the OECD 423-based design and the limited number of animals per dose group, the findings should be interpreted as exploratory and hypothesis-generating rather than statistically validated comparisons. In this screening model, the tested compounds showed distinct descriptive patterns of behavioral manifestations, peripheral organ involvement, and CNS findings. JWH-007 appeared to be associated mainly with depressant behavioral effects and peripheral organ toxicity; AM-694 showed limited observable behavioral manifestations despite evidence suggesting systemic organ involvement; and MAB-CHMINACA appeared to induce acute neurobehavioral signs together with CNS parenchymal alterations. These observations remain preliminary and require confirmation in adequately powered studies. Overall, the findings suggest that conventional GHS acute toxicity classification alone may not fully describe the biological effects observed for synthetic cannabinoids in exploratory preclinical models. Future hazard assessment may benefit from the inclusion of pharmacological, toxicokinetic, behavioral, biochemical, and neuropathological endpoints. The potential clinical and forensic relevance of these observations remains provisional and should be validated using larger experimental cohorts and clinical or postmortem datasets.

## Figures and Tables

**Table 1:** Behavioral manifestations after JWH-007 oral administration.

Monitored Parameter	50 mg/kg	300 mg/kg
Tremor	1/3 (0 → 15 min)	—
Straub tail	—	1/3 (30 min → 2 h)
Sedation	3/3 (15 min → 3 h), 1/3 (→4 h)	3/3 (15 min → 3 h), 1/3 (→4 h)
Excitation	1/3 (0 → 30 min)	—
Jumps	—	1/3 (0 → 15 min)
Motor incoordination	3/3 (0 → 2 h)	2/3 (0 → 30 min), 1/3 (→1 h)
Loss of balance	2/3 (0 → 30 min)	3/3 (0 → 15 min), 1/3 (→3 h)
Piloerection	—	1/3 (0 → 15 min)
Head twitches	1/3 (0 → 30 min)	—
Abnormal respiration	—	2/3 (15 min → 3 h)

Only effects observed in at least 1 of 3 animals are shown. Values indicate the number of affected animals/total number of administered animals, with the corresponding post-administration observation time interval given in parentheses (“—”—no observed effect, “→”—up to).

**Table 2:** Behavioral manifestations after JWH-007 i.p. administration.

Monitored Parameter	5 mg/kg	50 mg/kg
Sedation	3/3 (15 min → 2 h), 1/3 (→3 h)	3/3 (30 min → 4 h)
Motor incoordination	1/3 (0 → 1 h)	3/3 (0 → 15 min), 2/3 (→30 min)
Loss of balance	1/3 (0 → 1 h)	3/3 (0 → 30 min, 2 h), 1/3 (→3 h)
Seizures	—	3/3 (0 → 30 min), 1/3 (→1 h)
Tremor	—	1/3 (0 → 15 min)
Straub tail	—	2/3 (0 → 15 min)
Excitation	—	1/3 (0 → 15 min), 3/3 (15 min → 30 min)
Jumps	—	2/3 (0 → 15 min), 1/3 (15 min → 30 min)
Head twitches	—	2/3 (0 → 15 min)

Only effects observed in at least 1 of 3 animals are shown. Values indicate the number of affected animals/total number of administered animals, with the corresponding post-administration observation time interval given in parentheses (“—”—no observed effect, “→”—up to).

**Table 3:** Behavioral manifestations after MAB-CHMINACA oral administration.

Monitored Parameter	50 mg/kg	300 mg/kg
Seizures	3/3 (0 min → 1 h)	2/3 (0 → 15 min), 3/3 (→1 h), 1/3 (2 h)
Tremor	1/3 (0 → 15 min), 2/3 (→30 min, 1 h → 2 h), 1/3 (3 h)	1/3 (1 h)
Straub	1/3 (0 → 15 min), 2/3 (→30 min), 3/3 (30 min → 1 h), 1/3 (→2 h)	1/3 (1 h)
Sedation	1/3 (15 min), 2/3 (→30 min), 3/3 (1 h → 4 h)	2/3 (15 min, 30 min → 1 h), 1/3 (3 h → 4 h)
Excitation with jumps	3/3 (0 → 30 min)	1/3 (0 → 15 min)
Motor incoordination	2/3 (0 → 15 min), 3/3 (15 min → 1 h)	—
Loss of balance	3/3 (30 min)	
Writhes	2/3 (0 → 1 h), 1/3 (→2 h)	—
Loss of reactivity to touch	3/3 (0 min → 1 h)	—
Loss of grasping	1/3 (15 → 30 min)	—
Catalepsy	2/3 (15 → 30 min)	—
Loss of traction	1/3 (0 → 15 min), 2/3 (→30 min)	—
Abnormal respiration	1/3 (30 min)	

Only effects observed in at least 1 of 3 animals are shown. Values indicate the number of affected animals/total number of administered animals, with the corresponding post-administration observation time interval given in parentheses (“—”—no observed effect, “→”—up to).

**Table 4:** Behavioral manifestations after MAB-CHMINACA i.p. administration.

Monitored Parameter	5 mg/kg	50 mg/kg
Seizures	3/3 (0 min → 1 h), 2/3 (→2 h)	3/3 (0 min → 4 h)
Tremor	1/3 (0 → 15 min, 1 h)	2/3 (2 h)
Straub tail	1/3 (0 → 30 min), 2/3 (2 h)	2/3 (0 → 15 min), 1/3 (3 h)
Sedation	2/3 (2 h)	—
Excitation with jumps	3/3 (0 → 15 min), 2/3 (→30 min)	—
Motor incoordination	3/3 (0 → 15 min), 2/3 (→30 min)	
Head twitches	1/3 (0 → 30 min)	—
Loss of reactivity to touch	1/3 (0 → 15 min, 1 h)	—
Catalepsy	1/3 (0 → 15 min), 1/3 (30 min → 1 h)	—

Only effects observed in at least 1 of 3 animals are shown. Values indicate the number of affected animals/total number of administered animals, with the corresponding post-administration observation time interval given in parentheses (“—”—no observed effect, “→”—up to).

**Table 5:** Histopathological findings following acute i.p. exposure to SCs: AM-694, JWH-007, MAB-CHMINACA, in mice (n = 3 per group).

Group Dose/Route	Brain/CNS	Kidney	Liver	Pancreas	Spleen	Small Int.	Large Int.
Control							
0 mg/kg i.p.	None—A1	None—B1.	None—C1.	None—D1.	None—E1.	None—F1.	None—G1.
JWH-007							
5 mg/kg i.p.	None—A2	Vascular congestion (1/3)—B2.	Degeneration (1/3)—C2.	None—D2.	None—E2.	None—F2.	None—G2.
50 mg/kg i.p.	None—A2	Degeneration (3/3)—B3.	Degeneration (3/3); apoptosis, pyknotic nuclei (2/3)—C3.	Acinar changes with lipid inclusions (1/3)—D2.	None—E2.	None—F2.	Hypotrophy (1/3)—G2.
AM-694							
5 mg/kg i.p.	None—A4	Acidophilic nature of the proximal tubules (1/3)—B4.	None—C4.	None—D4.	None—E4.	None—F4.	None—G4.
50 mg/kg i.p.	None—A5	Vascular congestion (1/3)—B5.	Degenerative changes + apoptotic cells (1/3)—C5.	Hypertrophied Langerhans islets (1/3)—D5.	Megakaryocyte proliferation (2/3); stromal hyperplasia (1/3)—E5.	None—F5.	None—G5.
MAB-CHMINACA							
5 mg/kg i.p.	Small degenerative areas (1/3)—A6.	None—B6.	Focal necrosis (1/3) + pyknotic nuclei (1/3)—C6.	None—D6.	None—E6.	None—F6.	None—G6.
50 mg/kg i.p.	Small cerebral necrosis areas (2/3); vacuolar neuronal changes (2/3)—A7.	None—B7.	None—C7.	None—D7.	None—E7.	Edema + lymphoid infiltrates (2/3)—F7.	None—G7.

Values indicate the number of affected animals/total number of administered animals, in parentheses (“None”—No abnormalities). Alphanumeric grid coordinates (A–G, 1–7) denote the corresponding locations shown in Figure 3.

**Table 6:** Histopathological findings following acute oral exposure to SCs: AM-694, JWH-007, MAB-CHMINACA, in mice (n = 3 per group).

Group Dose/Route	Brain/CNS	Kidney	Liver	Pancreas	Spleen	Small Int.	Large Int.
Control							
0 mg/kg oral	None—A1.	None—B1.	None—C1.	None—D1.	None—E1.	None—F1.	None—G1.
JWH-007							
50 mg/kg oral	None—A2.	Vascular congestion (3/3)—B2.	Areas with pyknotic nuclei (1/3)—C2.	Hypertrophied islets; vacuolated serous cells (3/3)—D2.	Vascular congestion (2/3)—E2.	Edema + lymphoid infiltration (2/3)—F2.	None—G2.
300 mg/kg oral	None—A3.	None—B3.	Areas with pyknotic nuclei (1/3)—C3.	None—D3.	None—E3.	None—F3.	None—G3.
AM-694							
50 mg/kg oral	None—A4.	Acute tubular necrosis (2/3)—B4.	None—C4.	None—D4.	Megakaryocyte proliferation (2/3)—E4.	None—F4.	None—G4.
300 mg/kg oral	None—A5.	None—B5.	None—C5.	None—D5.	None—E5.	None—F5.	None—G5.
MAB-CHMINACA							
50 mg/kg oral	Cerebral necrosis (1/3)—A6.	Acute tubular necrosis (1/3)—B6.	Portal hypertension + focal hepatic necrosis (2/3)—C6.	None—D6.	Megakaryocyte proliferation (2/3)—E6.	None—F6.	None—G6.
300 mg/kg oral	Degenerated neurons (1/3)—A7.	None—B7.	None—C7.	None—D7.	Megakaryocyte proliferation (3/3)—E7.	None—F7.	None—G7.

Values indicate the number of affected animals/total number of administered animals, in parentheses (“None”—No abnormalities). Alphanumeric grid coordinates (A–G, 1–7) denote the corresponding locations shown in Figure 4.

## Data Availability

The datasets analyzed during the current study are available from the corresponding author upon reasonable request.

## Supplementary Materials

The following supporting information can be downloaded at: https://www.mdpi.com/article/10.3390/molecules31132365/s1.

## Abbreviations

The following abbreviations are used in this manuscript:

2-AG	2-arachidonoylglycerol
AEA	Anandamide
AM	Fluorinated benzoylindoles
AM-694	1-(5-fluoropentyl)-3-(2-iodobenzoyl) indole
BBB	Blood-brain barrier
CBD	Cannabidiol
CB1	Cannabinoid receptor 1
CB2	Cannabinoid receptor 2
CHMINACA	Indazole-carboxamides
CNS	Central nervous system
ECS	Human endocannabinoid system
GABA	Gamma-aminobutyric acid
GHS	Globally Harmonized System of Classification and Labelling of Chemicals
i.p.	Intraperitoneal
IVCs	Individually ventilated cages
JWH	Naphthoylindoles
JWH-007	1-pentyl-2-methyl-3-(1-naphthoyl) indole
LC-ESI-MS/MS	liquid chromatography–electrospray ionization tandem mass spectrometry
LD50	Median lethal dose
LOQ	Limit of quantification
MAB-CHMINACA	N-(1-amino-3,3-dimethyl-1-oxobutan-2-yl)-1-(cyclohexylmethyl)-1H-indazole-3-carboxamide
MRM	Multiple reaction monitoring
Na-CMC	Sodium carboxymethyl cellulose
NMDA	N-methyl-D-aspartate receptor
NPS	New psychoactive substances
OECD	Organisation for Economic Co-operation and Development
SCs	Synthetic cannabinoids
SEM	Standard error of mean
THC	Δ9-tetrahydrocannabinol
