# Supplementary material for: Acute Toxicity of Three Synthetic Cannabinoids: First In Vivo Preclinical Study
Source: Molecules. 2026 Jul 5;31(13):2365. doi: 10.3390/molecules31132365 (PMC13362526; doi:10.3390/molecules31132365)
Supplement: Supplementary file 1 [file molecules-31-02365-s001.zip › Suplementary_material.pdf]

Supplementary material

Acute Toxicity of Three Synthetic Cannabinoids: First In Vivo Preclinical Study

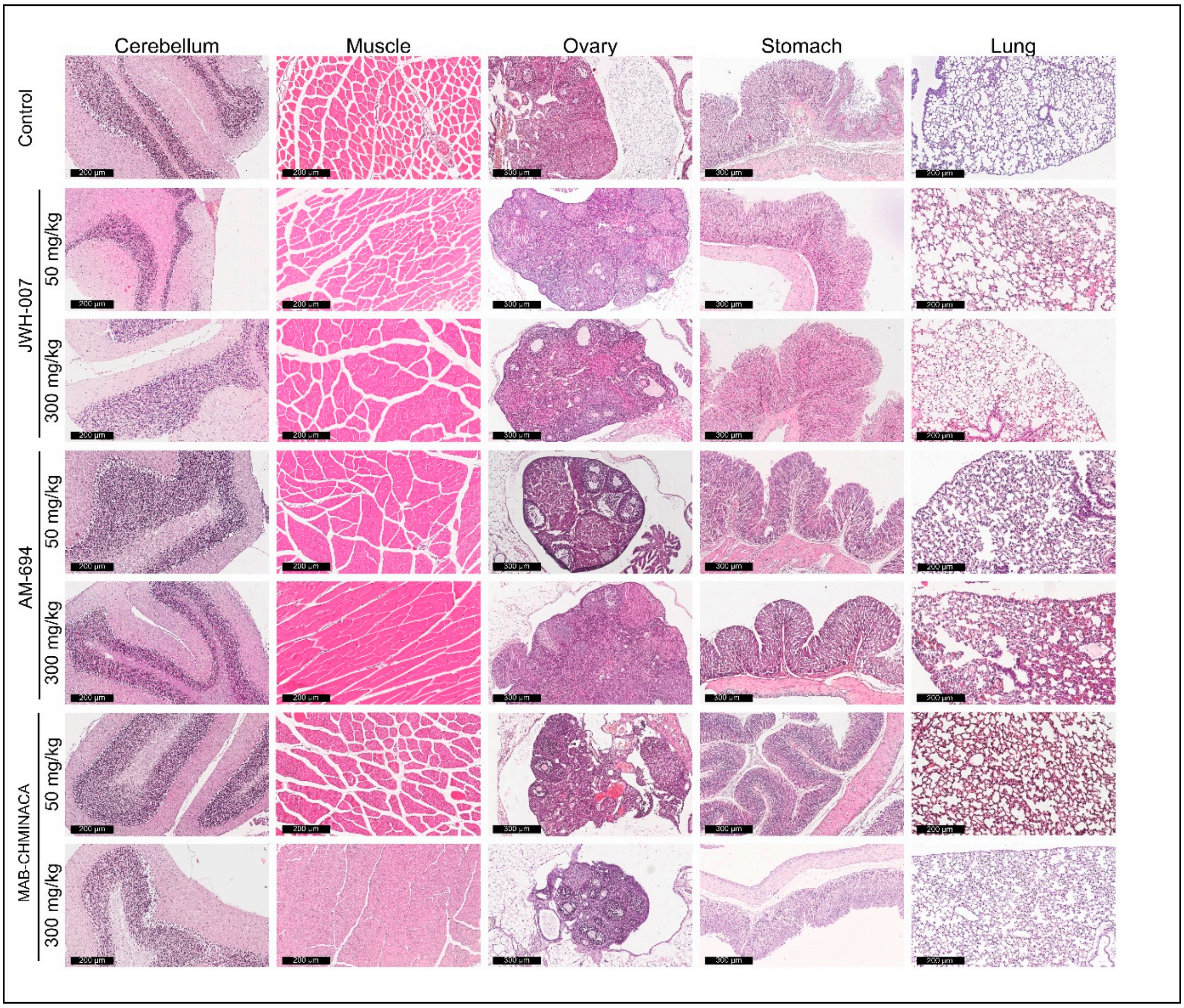

Figure S1. Histological examination of the cerebellum, spleen, skeletal muscles, ovary, stomach, lung in mice exposed to 50 mg/kg and 300 mg/kg of synthetic cannabinoids through oral administration with no morphological changes in all organs compared to the control group.

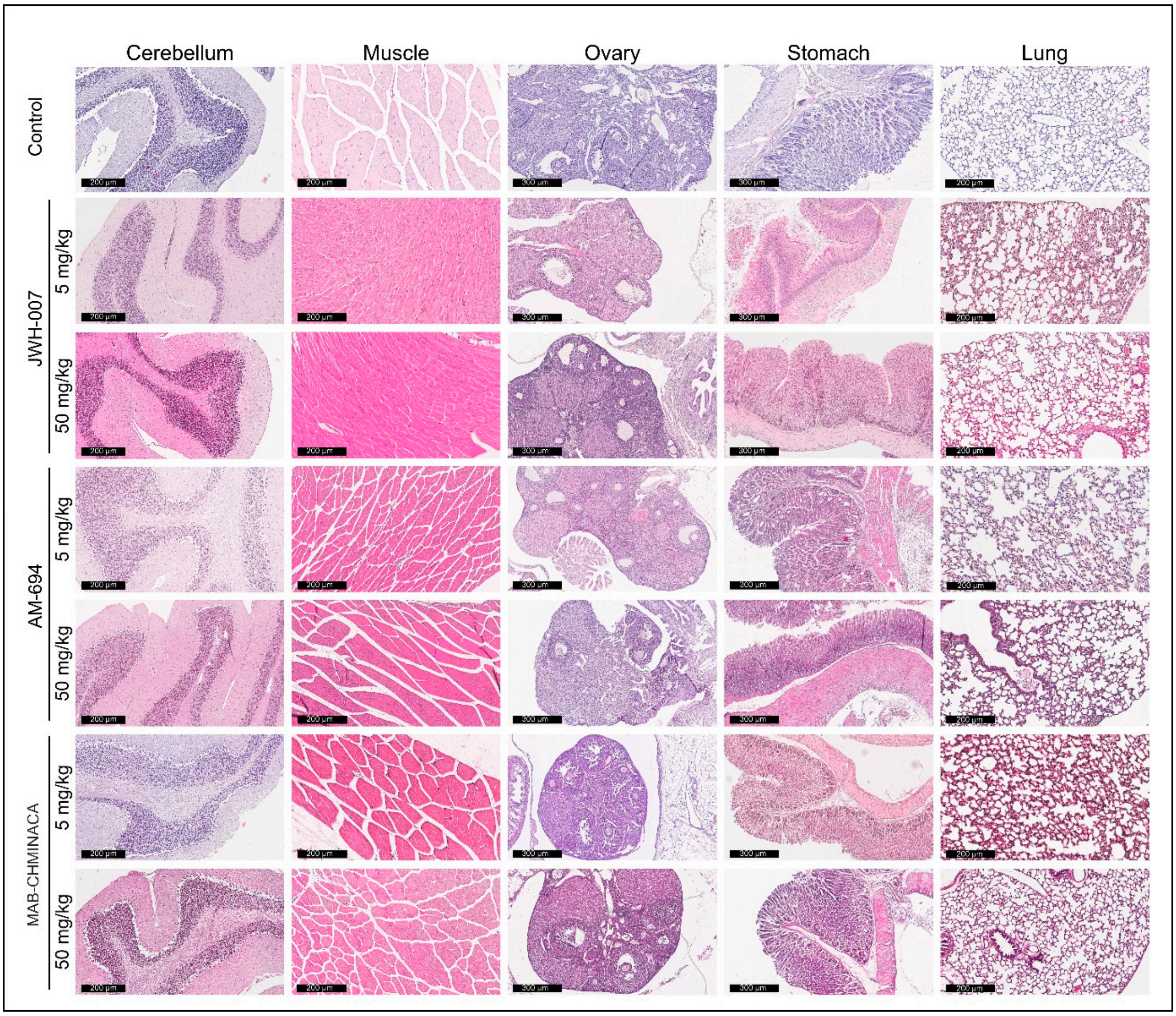

Figure S2. Histological examination of the cerebellum, spleen, skeletal muscles, ovary, stomach, lung in mice exposed to 50 mg/kg and 300 mg/kg of SCs through i.p. administration with no morphological changes in all organs compared to the control group, hematoxylin and eosin (H&E) standard staining protocol.

Table S1. Total organ injury score, mean ± SEM.

Group Dose/ Route	Brain / CNS	Kidney	Liver	Pancreas	Spleen	Small int.	Large int.
**Control**							
**0 mg/kg i.p.**	0	0	0	0	0	0	0
**0 mg/kg oral**	0	0	0	0	0	0	0
**JWH-007**							
**5 mg/kg i.p.**	0	0.67±0.67	0.67±0.67	0	0	0	0
**50 mg/kg i.p.**	0	2.67±0.33	3.67±0.88	0.67±0.67	0	0	1±1
**50 mg/kg oral**	0	3	0.67±0.67	4.67±0.33	1.67±0.88	3.33±1.76	0
**300 mg/kg oral**	0	0	0.67±0.67	0	0	0	0
**AM-694**							
**5 mg/kg i.p.**	0	0.67±1.15	0	0	0	0	0
**50 mg/kg i.p.**	0	0.67±1.15	2±2	1±1	2±1.15	0	0
**50 mg/kg oral**	0	2±1	0	0	1.33±0.67	0	0
**300 mg/kg oral**	0	0	0	0	0	0	0
**MAB-CHMINACA**							
**5 mg/kg i.p.**	0.33±1	0	1.67±0.88	0	0	0	0
**50 mg/kg i.p.**	1.33±2	0	0	0	0	2.67±1.33	0
**50 mg/kg oral**	0.67±0.67	1±1	3±1.53	0	1.67±0.88	0	0
**300 mg/kg oral**	1.33±0.67	0	0	0	2.33±0.33	0	0

Histopathological lesions were graded semi-quantitatively according to INHAND guidelines toxicologic pathology recommendations[1-6] using a five-point scale: 0 = no visible histopathological changes, 1 = minimal changes, 2 = mild changes, 3 = moderate changes, 4 = marked/severe changes [7]. Lesions were evaluated independently for each organ and animal by a histopathologist blinded to substances allocation. Organ-specific injury scores were subsequently calculated by summing the severity grades of all lesions identified within a given organ.
